# Supplementary material for: Human disturbances and the daytime activity of sympatric otters along equatorial Amazonian rivers
Source: PeerJ. 2023 Jul 21;11:e15742. doi: 10.7717/peerj.15742 (PMC10364808; doi:10.7717/peerj.15742)
Supplement: Supplemental Information 2 [file peerj-11-15742-s002.docx]

S2 Survey effort

Observational and telemetry studies show that activity of giant and neotropical otters is predominantly diurnal (Duplaix et al. 2015; Groenendijk et al. 2005; Rheingantz et al. 2017). In our study area the sun rises at approximately 06:30 am year round with day length ranging from 11.95 hours in December and 12.05 hours in June. Although effort was not distributed equally across all hours, our survey effort followed the expected activity patterns of both species and the recommended timing of river based boat surveys (Figure S2.1, (Groenendijk et al. 2005)).

|  |
| --- |
| 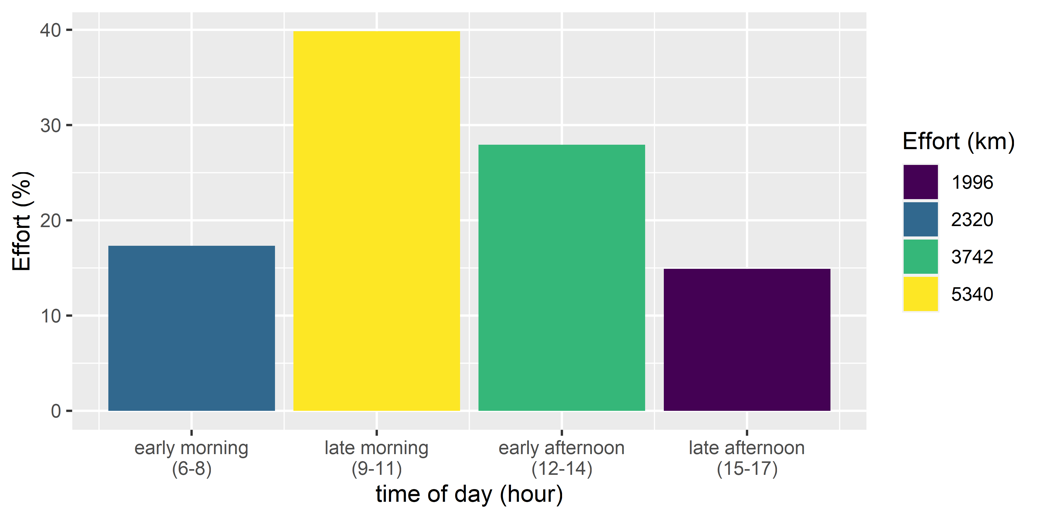 |

Figure S2.1 Distribution of survey effort by time of day.

There was greater survey effort (km per river reach, Fig. S2.2) in areas with more anthropogenic impacts, so any reduction in otter activity cannot be explained by differences in effort. There was more effort in reaches with than without nets (10,238 and 3161 km with and without nets respectively, Fig. S2.2). There was also less effort in reaches with few boats and no nets (1993 km) compared to many boats with nets (3619 km).

The presence of fishing nets and boat use in the river reaches were strongly correlated (Polychoric correlation rho = 0.84). The proportion of reaches with fishing nets increased with boat use, with 18%, 68% and 100% of reaches with fishing nets in the reaches with few, intermediate and many boats respectively (Fig. S2.2). This correlation meant that it would not be possible to include both nets and boats as covariates in the same model.


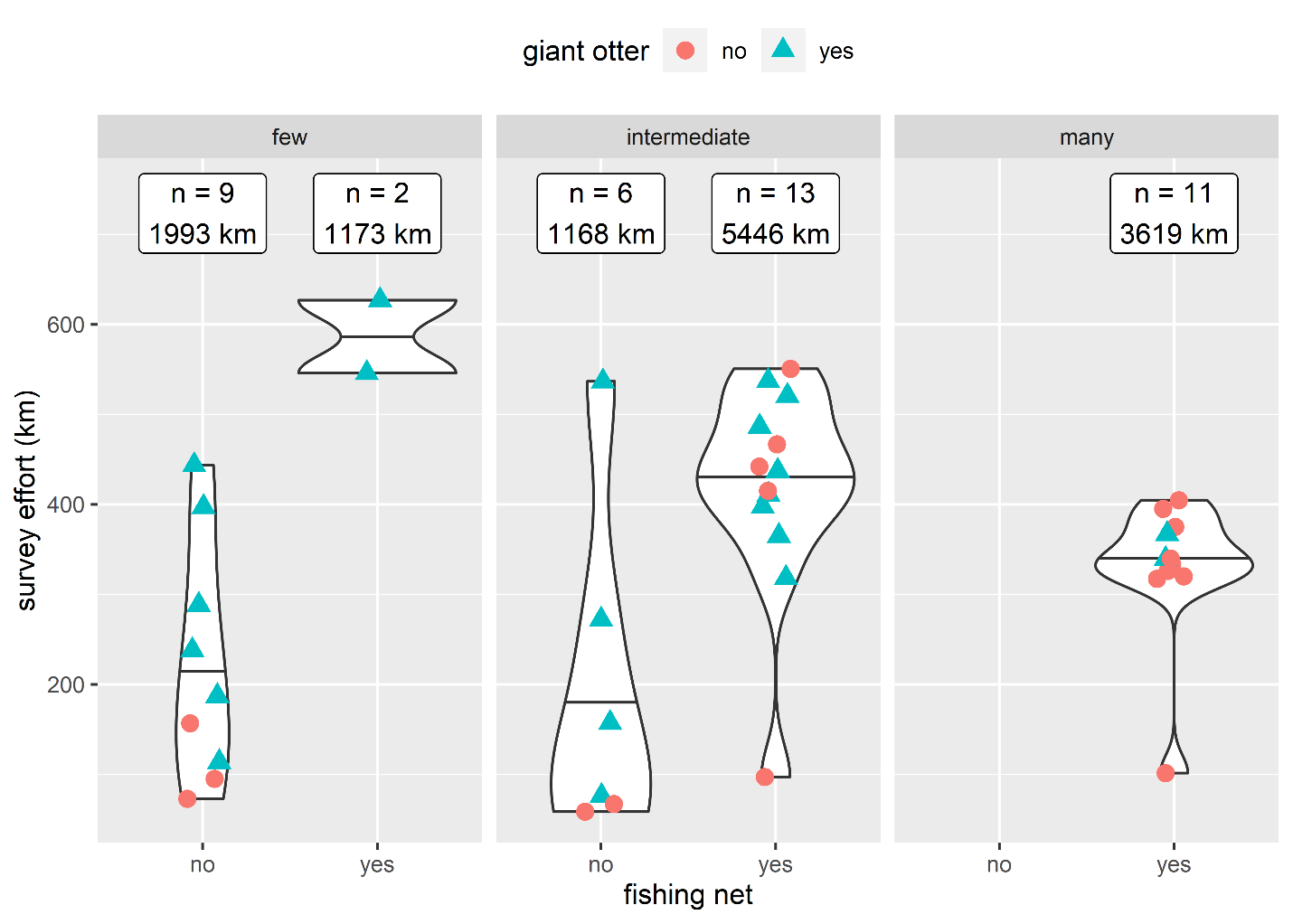


Figure S2.2. Distribution of survey effort. Points are from 41 river reaches classified according to human river use. Labels show number of reaches and km of survey effort. Violin plots with horizontal lines showing range and median values.

As we aimed to determine if patterns in neotropical otter activity changed due to the presence of the larger sized giant otter correlations were also calculated between the presence in reaches of giant otters, fishing nets and boats. The presence of giant otters was negatively correlated with both boats and nets (Polychoric correlation rho = -0.54 and -0.32 boats and fishing nets respectively). The proportion of reaches with giant otters decreased threefold from 67% in the least disturbed to 18% in most disturbed reaches (Fig S2.3). In contrast Neotropical otter encounters showed an opposite pattern with the proportion of reaches with neotropical otters nearly doubling from 44% in the least disturbed (few boats no nets) to 73% in most disturbed reaches (many boats with nets).

| 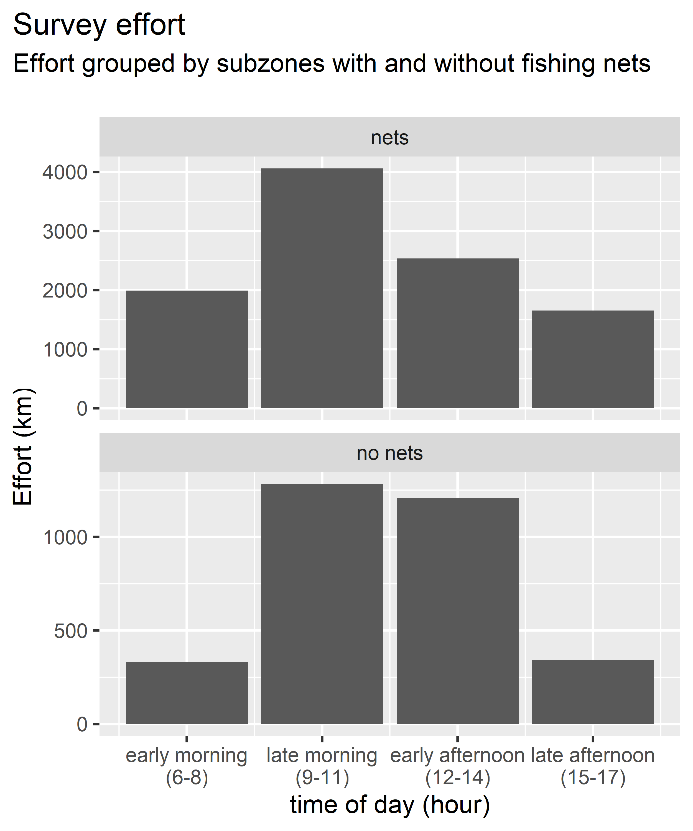 | 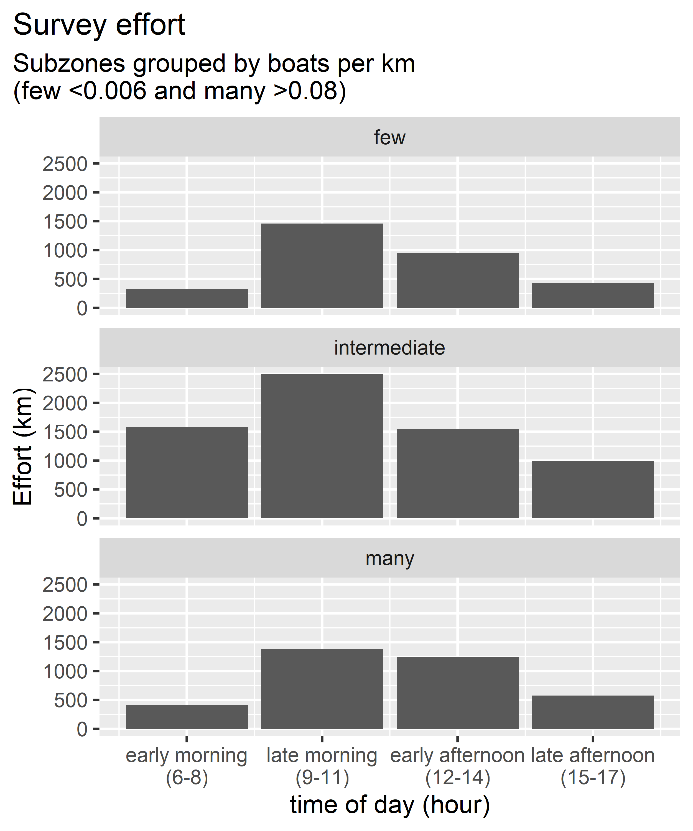 |
| --- | --- |

Figure S2.3. Distribution of survey effort by time of day and human river use.

References

Duplaix N, Evangelista E, and Rosas FC. 2015. Advances in the study of giant otter (Pteronura brasiliensis): ecology, behavior, and conservation: a review. *Latin American Journal of Aquatic Mammals* 10:75-98.

Groenendijk J, Hajek F, Duplaix N, Reuther C, Van Damme P, Schenck C, Staib E, Wallace R, Waldemarin H, and Notin R. 2005. Surveying and monitoring distribution and population trends of the giant otter (*Pteronura brasiliensis*): guidelines for a standardization of survey methods as recommended by the giant otter section of the IUCN/SSC Otter Specialist Group. *Habitat* 16:1-100.

Rheingantz ML, Santiago-Plata VM, and Trinca CS. 2017. The Neotropical otter Lontra longicaudis: a comprehensive update on the current knowledge and conservation status of this semiaquatic carnivore. *Mammal Review* 47:291-305. <https://doi.org/10.1111/mam.12098>
