# Supplementary material for: Human disturbances and the daytime activity of sympatric otters along equatorial Amazonian rivers
Source: PeerJ. 2023 Jul 21;11:e15742. doi: 10.7717/peerj.15742 (PMC10364808; doi:10.7717/peerj.15742)
Supplement: Supplemental Information 3 [file peerj-11-15742-s003.docx]

S3 Generalized Additive Models and Variable selection.

All models were run with the Binomial error family and estimated using restricted maximum likelihood (REML, (Pedersen et al. 2019; Wood 2017)). A combination of parametric, non-parametric smooths and interaction terms were employed to model the data (Table S3.1).

Table S3.1 Variables included to model temporal and spatial patterns.

|  | Variable | Term type | Term specification |
| --- | --- | --- | --- |
| Parametric |  |  |  |
|  | Boat frequency | Parametric factor with three levels. | boat |
|  | Net presence | Parametric factor with two levels. | net |
|  | Giant otter presence | Parametric factor with two levels. Only used to model neotropical otter activity. | pb |
| Spatial |  |  |  |
|  | Spatial - Geographic location | Non-parametric smooth term – tensor product interaction | te(lat, long, bs = c("tp", "tp"), k = c(12, 6), m = 2) |
|  | Spatio-temporal | Non-parametric smooth term – spatial tensor product interaction varies by season. | t2(lat, long, river_level, bs = c("tp", "tp", "re"), k = c(12, 6, 4), m = 2) |
| Temporal |  |  |  |
|  | Temporal – timing interacts with season | Non-parametric smooth term – tensor product interaction. Cyclic basis (“cc”) for the marginal season effect to account for the cyclic nature of seasonal river levels. | te(river_level, hour, bs = c("cc", "tp"), k = c(4, 6), m = 2) |
|  | Temporal – timing interacts with season and boats | Non-parametric smooth term – temporal tensor product interaction varies by boat frequency. | t2(river_level, hour, boat, bs = c("cc", "tp", "re"), k = c(4, 6, 3), m = 2, full = TRUE) |
|  | Temporal – timing interacts with season and nets | Non-parametric smooth term – temporal tensor product interaction varies by net presence. | t2(river_level, hour, net, bs = c("cc", "tp", "re"), k = c(4, 6, 2), m = 2, full = TRUE) |
|  | Temporal – timing interacts with season and giant otters | Non-parametric smooth term – temporal tensor product interaction varies by giant otter presence. Only used to model neotropical otter activity. | t2(river_level, hour, pb, bs = c("cc", "tp", "re"), k = c(4, 6, 2), m = 2, full = TRUE) |
| Effort | |  |  |
|  | Effort | Offset | offset(effort) |
|  |  |  |  |

Table S3.2 Global model results. Estimates and summaries for global Generalized Additive Models used to explain patterns in otter activity.

|  | | |  | Giant otter | | |  | Neotropical otter | | |
| --- | --- | --- | --- | --- | --- | --- | --- | --- | --- | --- |
| Parametric | | |  | Estimate | SE | P |  | Estimate | SE | P |
|  | intercept | | | -14.63 | 22.03 | 0.507 |  | 28.58 | 35.95 | 0.427 |
|  | boat | | |  |  |  |  |  |  |  |
|  | intermediate vs few | | | 1.87 | 2.09 | 0.370 |  | -5.27 | 10.97 | 0.631 |
|  | many vs few | | | -21.90 | 12.87 | 0.089 |  | -32.27 | 12.92 | 0.013 |
|  | net - with vs without | | | 5.65 | 4.85 | 0.244 |  | -3.94 | 7.82 | 0.614 |
|  | Pb - with vs without | | | - | - | - |  | -1.73 | 3.70 | 0.640 |
| Spatial | | |  | EDF |  | P |  | EDF |  | P |
|  | Geographic location | | | 24.72 |  | 0.714 |  | 21.85 |  | 0.998 |
|  | Spatio-temporal | | | 22.73 |  | 0.442 |  | 38.23 |  | 0.697 |
| Temporal | | |  | EDF |  | P |  | EDF |  | P |
|  | Timing interacts with season | | | 11.66 |  | 0.664 |  | 9.32 |  | 0.427 |
|  | Timing interacts with season and boats | | | 12.43 |  | 0.109 |  | 20.52 |  | 0.098 |
|  | Timing interacts with season and nets | | | 15.97 |  | 0.194 |  | 9.57 |  | 0.307 |
|  | Timing interacts with season and giant otters | | | - |  | - |  | 8.27 |  | 0.849 |
| Model | | |  |  |  |  |  |  |  |  |
|  | | Observations | |  | 1296 |  |  |  | 1296 |  |
|  | | Deviance explained (%) | |  | 43.2 |  |  |  | 46.2 |  |
|  | | QAIC | |  | 491.7 |  |  |  | 476.4 |  |

EDF: Estimated degrees of freedom for the model terms. Values close to zero indicate no relationship with the response, close to 1 may suggest a linear relationship and values greater than 1 suggest a non-linear relationship.

Model deviance explained. (%): Percent of total deviance explained.

QAIC: Quasi- Akaike Information Criterion.

References

Pedersen EJ, Miller DL, Simpson GL, and Ross N. 2019. Hierarchical generalized additive models in ecology: an introduction with mgcv. *PeerJ* 7:e6876. 10.7717/peerj.6876

Wood SN. 2017. *Generalized Additive Models: An Introduction with R, Second Edition (2nd ed.)*. Boca Raton: Chapman and Hall/CRC.
